# Unanimo Consensu

**Published:** 1879-12

**Authors:** Herman Mynter


### UNANIMO CONSENSU.

TRANSLATED FROM THE DANISH BY HERMAN MYNTER, M. D.

THREE medical professors in Lisbon, Bento de Sause, Martins and Cabral, have published an account of a celebrated case of an exhumed corpse, to decide whether the deceased had hung himself or had been strangled. The three experts mentioned declared for the latter alternative, but found ardent opponents in three members of the faculty of Coimbra, according to whose declaration the jury acquitted the defendant.

The professors, at Lisbon, applied in a joint letter to experts in medical jurisprudence in all countries of Europe, gave them the matter at issue, and asked them to give their opinion. The physicians asked were: Hoffman, of Vienna; Aloys Martin, of Munich; Guillery, of Brussels; Gaedeken, of Copenhagen; Bergeron and Tardieu, of Paris; Pallis, Geonganlas and Arphanides, of Athens; y Vivo, of Barcelona; y Mendoza, of Cadiz; Yanes, of Madrid; de Myer, of Utrecht; Bristowe, Crospy and Taylor, of London; Romati, of Bologna; Lazzaretti, of Padua; Pacchiotti, of Turin; Herberg, of Christiania; Mando and Pinto, of Coimbra; Vianna and Pitta, of Lisbon; Osonio, of Ponto; Liman, of Berlin; Tchitowitsh and Lenz, of St. Petersburg; Jaederholm, of Stockholm; Gurmert, of Bern. The answers from all these experts agreed that the party concerned "must be considered to have been strangled." Such an unanimity is both interesting and of importance, proving the high development of the science of forensic medicine.—*Hospitals-Fidenden.*

